# Supplementary material for: Does the Element Availability Change in Soils Exposed to Bioplastics and Plastics for Six Months?
Source: Int J Environ Res Public Health. 2022 Aug 4;19(15):9610. doi: 10.3390/ijerph19159610 (PMC9368576; doi:10.3390/ijerph19159610)
Supplement: Supplementary file 1 [file ijerph-19-09610-s001.zip › ijerph-1809658-supplementary.pdf]

Table S1. Mean values (± s.e.) of Total Al, Ca, Cu, Fe, K, Mg, Mn, Na Pb and Zn (expressed as mg g^{-1} d.w.), Ni (expressed as μg g^{-1} d.w) concentrations and available Ca, K, Mg and Na (expressed as mg g^{-1} d.w.), and Al, Cu, Fe, Mn, Ni, Pb and Zn (expressed as μg g^{-1} d.w) measured in soils at the beginning of experiment (T0).

	**Total**	**Available**
Al	66.5	2.07
Ca	38.2	5.56
Cu	0.135	47.5
Fe	30.6	38.9
K	33.8	1.80
Mg	9.53	0.433
Mn	0.870	8.12
Na	4.41	0.875
Ni	21.1	0.285
Pb	0.1	9.02
Zn	0.183	32.1
